# Nonpharmacological interventions to reduce short-term or long-term psychological stress in ICU patients: a systematic review

**DOI:** 10.1186/cc14636

**Published:** 2015-03-16

**Authors:** D Wade, Z Moon, S Windgassen, J Weinman

**Affiliations:** 1 University College Hospital, London, UK; 2 Kings College London, UK

## Introduction

A systematic review was performed of studies of nonpharmacological interventions aiming to reduce short-term or long-term stress in intensive care patients, as little is known about the efficacy of such interventions. Previous work has shown that intensive care patients undergo many stressful experiences, which can affect their long-term psychological well-being. Studies have demonstrated a high prevalence of depression, anxiety or post-traumatic stress disorder after intensive care admissions.

## Methods

A systematic review was carried out according to the Prisma statement. A search was conducted of Medline, Embase and Psychinfo databases. Inclusion criteria included studies of populations of adult patients in mixed or general ICUs. No study designs were excluded, but studies that focused on specific disease states were excluded. Included studies were assessed for risk of bias, using a quality checklist.

## Results

A total of 1,743 papers were retrieved, of which 18 studies were eligible for inclusion in the review. Studies had a combined population of 1,970 patients admitted to 38 ICUs from Europe, Asia and North America. Eleven studies were randomized controlled trials (RCTs). Interventions were classified as four groups - music; therapeutic touch; diary and psychotherapeutic interventions. Ten studies found that music interventions were effective in the short term; however, follow-up results were limited and some studies were low quality. There was moderate quality evidence from three studies for the effectiveness of diary interventions, with medium-term follow-up results. There was mixed-quality evidence for therapeutic touch interventions in the short term from three studies. The two psychotherapeutic interventions studied were of moderate quality, and one showed promising results at 12-month follow-up.

## Conclusion

The evidence for the efficacy of nonpharmacological interventions to reduce short-term or long-term stress in intensive care patients was of low to moderate quality. Studies included mainly short-term and medium-term follow-up. This highlights the need for larger-scale, better-quality RCTs with longer-term outcome measurement. However, the results indicate that nonpharmacological, including psychological, approaches are likely to be beneficial for reducing short-term or long-term stress in intensive care patients.

